# Very late relapse of high-grade osteosarcoma: A case report and review of the literature

**DOI:** 10.1097/MD.0000000000021206

**Published:** 2020-07-17

**Authors:** Yoichi Kaneuchi, Michiyuki Hakozaki, Hitoshi Yamada, Osamu Hasegawa, Shoki Yamada, Yuka Oka, Kazuo Watanabe, Shinichi Konno

**Affiliations:** a Department of Orthopaedic Surgery; b Department of Radiology; c Department of Pathology and Diagnostic Pathology, Fukushima Medical University School of Medicine; d Fukushima Pathology Laboratory, Fukushima, Japan.

**Keywords:** metastasis, osteosarcoma, recurrence

## Abstract

**Rationale:**

Osteosarcoma is the most common primary malignant bone tumor in children. The prognosis of osteosarcoma has improved with the use of aggressive systemic chemotherapy in addition to surgery. The relapse of osteosarcomas is usually as lung metastasis observed within 2 to 3 years after the initial treatment. A relapse is rarely observed at >10 years.

**Patient concerns:**

We report the case of a 51-year-old Japanese man who was treated for high-grade osteosarcoma of the femur at 13 years old. He was referred to our hospital with a suspicion of primary lung cancer based on back pain, respiratory distress, and an abnormal mass on chest radiograph.

**Diagnoses:**

Computed tomography-guided biopsy confirmed the lung lesion as a metastatic recurrence of high-grade osteosarcoma without local recurrence.

**Interventions:**

Chemotherapy was planned, but the patient's general condition rapidly deteriorated and thus palliative therapy was provided.

**Outcomes:**

The patient died 2 months after the initial consultation.

**Lessons:**

The survival durations of osteosarcoma patients have been prolonged by recent progress in multimodality therapy, and thus clinicians as well as osteosarcoma patients should always keep in mind the possibility of very late relapse.

## 1 Introduction

The prognosis of osteosarcomas has improved with the use of aggressive systemic chemotherapy in addition to surgery.^[1,2]^ Despite the use of multimodality therapy, 30% to 50% of osteosarcoma patients with no metastasis at diagnosis develop local or metastatic recurrence.^[3,4]^ The lung is the most common metastatic site, and pulmonary metastasis typically occurs within 2 to 3 years after initial treatment.^[5–7]^ Relapse occurring ≥10 years after an initial treatment for osteosarcoma is exceedingly uncommon and has been described in few reports.^[5,8–15]^ We describe an extremely rare case of osteosarcoma that recurred as lung metastasis 38 years after the patient's initial treatment with surgery and chemotherapy. Ethical approval was waived by the institutional review board because this study is a case report. Written informed consent was obtained from the bereaved family of patient for publication of this case report with accompanying images.

## 2 Case presentation

**Figure 1:**
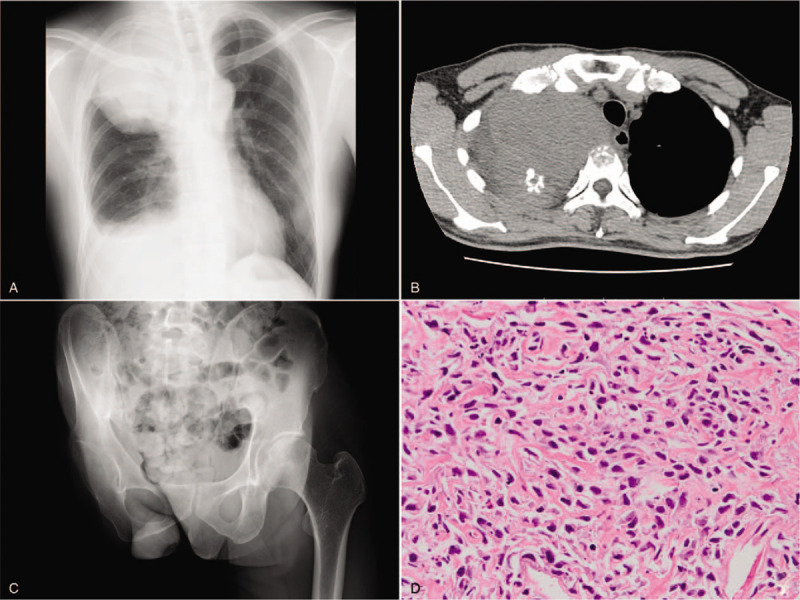
Plain chest radiograph reveals a large tumoral mass in the right upper lung field with pleural effusion (A). Whole-body CT showing a large tumor with calcification and/or ossification occupying the apex area of the right lung (B). There is no sign of local recurrence on a plain radiograph of the hip joint (C). Histopathological examination of the biopsy specimen shows a proliferation of atypical pleomorphic tumor cells producing lace-like osteoid tissue (hematoxylin-eosin stain, high-power field) (D). CT = computed tomography.

A 51-year-old Japanese man was referred to our hospital with a 3-month history of right back pain and respiratory distress. At the age of 13 years, he had been diagnosed with high-grade osteosarcoma of the right femur and underwent hip disarticulation followed by adjuvant chemotherapy at a different hospital. Since there was no sign of local or metastatic recurrence at 2 years after that treatment, the patient stopped visiting the hospital. At his initial visit to our hospital, chest plain radiographs revealed a large tumoral mass with pleural effusion in the right upper lung field (Fig. 1A). Primary lung cancer was suspected. Whole-body computed tomography (CT) showed a large tumor with mineralization occupying the apex area of the right lung (Fig. 1B). Small nodules scattered in the bilateral lung field, pleural effusion, bone metastases to the thoracic spine and ribs, and bilateral adrenal metastasis were detected. An additional hip radiograph showed no evidence of local recurrence (Fig. 1C).

The laboratory findings indicated hyperphosphatasemia (serum alkaline phosphatase: 668 IU/L, normal range 115–359 IU/L). Regarding the serum tumor markers, sialyl Lewis X-i was modestly elevated (45 U/mL, normal range <38 U/mL), but the results for cytokeratin 19 fragment and pro-gastrin-releasing peptide were negative. To obtain a definitive diagnosis, a CT-guided transthoracic needle biopsy for the lung tumor was performed. The histopathological examination showed a proliferation of atypical pleomorphic tumor cells producing lace-like tumoral osteoid tissue (Fig. 1D). Immunohistochemical stainings for AE1/AE3, desmin, epithelial membrane antigen, and S-100 protein were negative, whereas those for vimentin, smooth muscle actin, and special AT-rich sequence-binding protein 2 were positive. We diagnosed the lung tumor as a metastatic recurrence of osteosarcoma.

Although chemotherapy was planned, the patient's general condition rapidly deteriorated, and he died 2 months after the initial consultation.

## 3 Discussion

Late relapse of osteosarcoma is defined as local or metastatic recurrence at ≥5 years after initial treatment.^[5,11,14]^ In low-grade central osteosarcoma cases, the incidence rate of late relapse was relatively high, and it occurred in 5.5% to 30% of the cases.^[16–19]^ In contrast, late relapse of high-grade osteosarcoma is rare, with an incidence of 0.6% to 2.9% (Table 1).^[1,2,5,9,10,20]^ Very late relapse, defined as local or metastatic recurrence at ≥10 years after initial treatment, rarely occurs (0%–0.4%) in high-grade osteosarcoma cases.^[1,2,5,9,10,20]^ There are 6 studies on late relapse and these included reports from the Memorial Sloan-Kettering Cancer Center (MSKCC), a Cooperative Osteosarcoma Study Group (COSS-86) analysis, a COSS intergroup analysis, the London Bone and Soft tissue Tumour Service, the Rizzoli Institute, and Hauben study of a total 2243 patients including patients treated by the European Osteosarcoma Intergroup (EOI), the COSS intergroup, and the Royal Orthopaedic Hospital (ROH) in Birmingham.

**Table 1:**
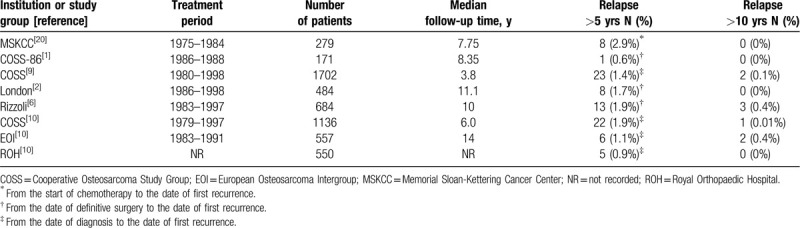
Published data on the incidence of late relapse in patients with high-grade osteosarcoma.

In the MSKCC analysis of 279 patients with localized osteosarcoma followed-up for a median of 7.75 years, 8 patients (2.9%) developed late relapse.^[20]^ The COSS-86 analysis of 171 osteosarcoma patients treated between 1986 and 1988 and followed for a median of 8.35 years showed that only 1 late relapse (0.6%) occurred.^[1]^ In the data from the COSS analysis of 1702 patients treated between 1980 and 1998 with a median follow-up of 3.8 years, 23 (1.4%) and 2 (0.1%) patients developed recurrence after 5 and 10 years after their diagnoses, respectively.^[9]^ The London experience demonstrated a late relapse incidence of 1.7%: 8 of 484 osteosarcoma patients treated between 1986 and 1998 with a median follow-up of 11.1 years.^[2]^ The data from the Rizzoli Institute showed that 13 (1.9%) of 684 patients developed late relapse and only 3 (0.4%) patients experienced late recurrence after 10 years.^[5]^ Hauben et al^[10]^ reported their analysis of patients from 3 groups (the COSS, EOI, and ROH), and the patient inclusion criteria in the analysis were age <40 years old at the diagnosis, with a high-grade osteosarcoma of an extremity, no metastasis at diagnosis, no history of other primary malignancy, and no prior treatment with chemotherapy or radiotherapy. In the data from the COSS analysis obtained in patients treated between 1979 and 1997 with a median follow-up of 6.0 years, 22 (1.9%) of 1136 patients developed late relapse and only 1 (0.01%) patient experienced very late relapse. The analysis of EOI patients treated between 1983 and 1991 described the incidence rates of late relapse of 1.1% (6 of 557 patients) and very late relapse at 0.4%. The ROH experience of 550 patients showed that only 5 (0.9%) patients developed late relapse.

Very late relapse is thus extremely rare. To the best of our knowledge, only 17 cases of high-grade osteosarcoma, including the present case, have been published as a case report or part of a case series or large cohort study (Table 2).^[5,8–15]^ Strauss et al^[2]^ reported the case of a patient with metastatic relapse 14 years after initial treatment. However, that patient developed a first lung metastasis at the end of adjuvant chemotherapy and underwent a pulmonary metastasectomy, and we therefore excluded the patient from the group of very late relapse cases described herein. The patterns of very late relapse in the 17 high-grade osteosarcoma cases were as follows: distant metastasis in 13 patients (including 11 pulmonary metastases, 1 bone metastasis, 1 both bone and pulmonary metastases) and local recurrence in 4 patients. Among the 16 previously reported patients, the longest disease-free interval between initial treatment and lung metastatic recurrence was 27 years,^[11]^ and that for local recurrence was 19.3 years.^[5]^ Only 3 patients developed lung metastases >20 years after their initial treatment.^[5,8,14]^ Our patient's very late relapse was identified 38 years after his initial treatment, and this is the longest disease-free interval of high-grade osteosarcoma reported in the English literature.

**Table 2:**
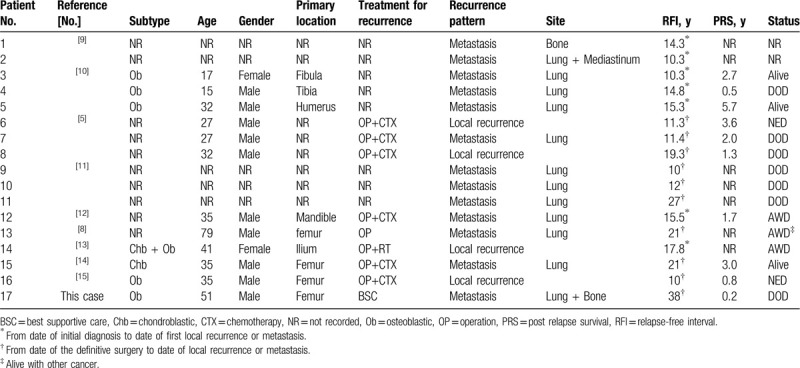
Clinical characteristics of patients with very late relapse of high-grade osteosarcoma.

## 4 Conclusion

The present patient's case is quite instructive because it represents the longest disease-free interval of a high-grade osteosarcoma. Since the survival duration of osteosarcoma patients have been prolonged by recent progress in multimodality therapy, clinicians as well as osteosarcoma patients should always keep in mind the possibility of very late relapse.

## Author contributions

**Conceptualization:** Yoichi Kaneuchi, Michiyuki Hakozaki.

**Investigations:** Shoki Yamada, Yuka Oka, Osamu Hasegawa.

**Methodology:** Hitoshi Yamada.

**Visualization:** Kazuo Watanabe.

**Supervision:** Hitoshi Yamada.

**Writing - original draft:** Yoichi Kaneuchi.

**Writing – review & editing:** Michiyuki Hakozaki, Shinichi Konno.
